# Basic immunologic study as a foundation for engineered therapeutic development

**DOI:** 10.1002/prp2.1168

**Published:** 2024-06-18

**Authors:** Sabrina DeStefano, Daphna Fertil, Mondreakest Faust, Kaitlyn Sadtler

**Affiliations:** ^1^ Section on Immunoengineering, National Institute of Biomedical Imaging and Bioengineering National Institutes of Health Bethesda Maryland USA

**Keywords:** immunology, regeneration, tissue, wound healing

## Abstract

Bioengineering and drug delivery technologies play an important role in bridging the gap between basic scientific discovery and clinical application of therapeutics. To identify the optimal treatment, the most critical stage is to diagnose the problem. Often these two may occur simultaneously or in parallel, but in this review, we focus on bottom‐up approaches in understanding basic immunologic phenomena to develop targeted therapeutics. This can be observed in several fields; here, we will focus on one of the original immunotherapy targets—cancer—and one of the more recent targets—regenerative medicine. By understanding how our immune system responds in processes such as malignancies, wound healing, and medical device implantation, we can isolate therapeutic targets for pharmacologic and bioengineered interventions.

## Graphical abstract

Multiple phases in wound healing and tissue regeneration have immune cell involvement and are targets for pharmacologic intervention. Understanding these pathways can yield insight into best treatment approaches and delivery routes.

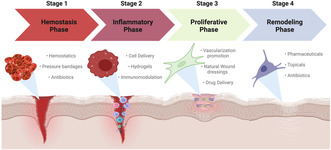

**Abbreviations**
ATP: Adenosine triphosphate
CAR: Chimeric antigen receptor
CTLA4: cytotoxic T‐lymphocyte associated protein 4
DC: Dendritic cells
DFU: Diabetic foot ulcer
ECM: Extracellular matrix
FBR: Foreign body response
HMGB1: High mobility group box 1 protein
HSV: Herpes simpelx virus
IFNg: Interferon gamma
IL: Interleukin
mTOR: Mammalian target of rapamycin
NK Cell: Natural Killer Cell
PD1: programmed cell death 1
ROS: Reactive oxygen species
STING: stimulator of interferon genes
TLR: Toll‐like Receptor
TNF: Tumor necrosis factor
VEGF: Vascular endothelial growth factor

## 1 MAIN TEXT

Basic biology, clinical medicine, and bioengineering work in concert to identify the molecular mechanisms of how our cells and bodies function during health and disease, and to determine downline systems to model and treat diseases.^1^ Engineering and pharmacology can be employed to translate basic biologic understanding into clinical therapeutics. Furthermore, engineering technologies can also be used to further our understanding of basic biology. In this review, we will discuss advances of this intersection of basic biology and bioengineering through the lens of two fields: cancer therapeutics and regenerative medicine. Though seemingly disparate these two fields are linked by a common thread—immunologic self‐tolerance and self‐reactivity to yield either pathogenic or pro‐healing effects. This can be seen with prior mechanistic studies that implicate similar pathways both in response to tumors and response to tissue injury and material implantation.^2^, ^3^, ^4^ Through leveraging these similarities, it is possible to learn from cancer immunology and regenerative medicine to yield insights that help therapeutics with both fields.

### 1.1 Cancer immunoengineering

Cancer has been a principal cause of morbidity and mortality worldwide for many years. Throughout the body, cells become cancerous and begin to proliferate uncontrollably. This process can be triggered by unregulated and or altered genes that control cell division, leading to tumor formation. Tumors themselves are immunomodulatory, which reduces the immune system's ability to recognize and destroy cancer cells allowing them to grow on almost any tissue or organ in the body. The survival rate and quality of life of cancer patients have gradually increased due to the availability of advanced treatments.

#### 1.1.1 Beginnings of cancer immunotherapy: bacteria and Coley's toxin

In 1898, Dr. William B. Coley, the inventor of immunotherapy began experimenting with a bacterial cocktail that had the ability to eradicate malignant cells from the human body.^5^ This finding led to the discovery of utilizing a therapeutic cocktail containing live and inactivated *Streptococcus pyogenes* and *Serratia marcescens* to treat patients with bacterial infections and eradicate their tumors. Some types of cancer cells were successfully destroyed; however, the mechanistic method of the cocktail was not known which led to scientists speculating that this may have been caused by an incredibly strong immune response that overwhelmed the cancer cells. Coley's toxins were utilized in hospitals for several years, but the side effects were always unpredictable. The heterogeneity of these materials, uneven dosing, uncertain pharmacokinetics, and unknown mechanism of action made it prohibitive to utilize these toxins in the clinic. Eventually, a policy was formed that prohibited the use of toxins, discouraging the use of immunotherapy, and led to the use of chemotherapy causing a long‐term decrease in the interest of immunotherapeutic approaches. In the mid‐1900s, Coley's work was revived due to the discovery of T cells made by Thomas and Burnet. They proposed that T cells were surveilling the body specifically for tumor‐associated antigens and their respective proteins upon transforming into malignant cells to tag them as cancerous and “abnormal” for clearance by the immune system.^6^ The mechanism of “Coley's toxin” is now known to be the inflammatory activation of the immune system via bacterial toxins, thereby overwhelming the immunoregulatory nature of tumors. In addition to bacterial toxins, researchers have since found that oncolytic viruses can also cause damage to tumors and immune activation against these transformed cells.

#### 1.1.2 Engineered viruses for cancer therapeutics

**FIGURE 1:**
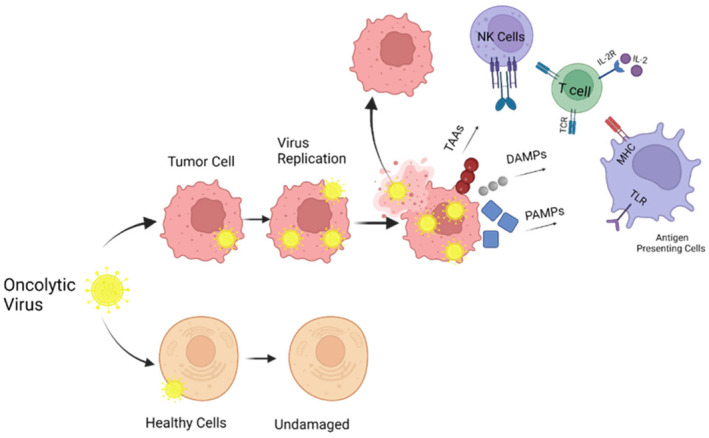
Mechanism of action for oncolytic virus activation. This occurs in the presence of TLR and pathogen‐associated molecular patterns further activating the immune system against tumor cells. Either the oncolytic virus targets healthy cells leaving the cell undamaged or it targets tumor cells further replicating the virus within the cell causing IL‐2 and TNF‐⍺ to stimulate immune cells such as NK and T cells to respond to the cancerous cell leading to necrosis or apoptosis. Modified from “Cancer Mechanisms,” by BioRender.com (2023). Retrieved from BioRender.com.

The general mechanism of action for oncolytic viruses is through the activation of toll‐like receptors (TLR) in the presence of pathogen‐associated molecular patterns. Consequently, inflammatory molecules such as the interleukin 2 (IL‐2) and tumor necrosis factor alpha (TNF‐⍺) are released, simulating cancer death through necrosis or apoptosis.^7^, ^8^ Release of IL‐2 has been linked to the maturation and expansion of CD8+ cytotoxic T lymphocytes and natural killer cells.^7^ As tumor cells die, damage‐associated molecular patterns such as ATP, HMGB1, and uric acid are released, simulating inflammatory cytokine production.^9^ Tumor cell characteristics such as the over expression of viral receptors, a proliferative cell cycle and cell deficiencies in molecules such as interferon, facilitate selective infection of cells. A modified type 1 herpes simplex virus (HSV); T‐VEC—an attenuated virus with a variety of gene deletions and insertions to enhance antitumor immune response, in 2015 became the first FDA‐approved oncolytic virus to be used for unresectable stage III advanced melanoma.^8^ Patients treated with T‐VEC showed a lower level of immune‐suppressive cells such as CD4+ FoxP3+ regulatory T cell compared to the untreated group. The continuance of pre‐existing immunity and the inability to provide direct injection to the tumor area are two factors that may affect the efficiency of oncolytic HSV therapeutics (Figure 1).

#### 1.1.3 Checkpoint blockade immunotherapy

By further evaluating how immune cells regulate their activation to inflammatory stimuli, such as those induced by bacterial toxins and oncolytic viruses, researchers were able to determine how tumors inhibited immune cell activation, thereby generating new immunotherapies. Drs. James Allison and Tasuku Honjo, discovered cytotoxic T‐lymphocyte‐associated protein 4 (CTLA‐4) and programmed cell death 1 (PD‐1) role as immune checkpoint inhibitors, which has transformed cancer therapy.^10^ CTLA‐4 is a cell‐surface protein found on T cells that when bound to ligands CD80 (B7–1) and CD86 (B7–2) inhibits the activation of T cells. Ipilimumab was the first anti‐CTLA‐4 monoclonal antibody (mAb) approved for human use in 2011 for the treatment of late‐stage melanoma that was difficult to treat with surgery.^11^ This blocking antibody prevents engagement of CTLA‐4, which ultimately prevents tumor‐mediated suppression of T cell activation. As with CTLA‐4, PD‐1 inhibits T cell activation when bound to PD‐L1 and PD‐L2 ligands. Engineering a blocking antibody against PD‐1 lead to the creation of the FDA‐approved anti‐PD‐1 therapeutic for metastatic melanoma in 2014. The creation of these blocking antibody therapeutics was not the only development in the field as an outcome of these basic biologic studies; recently CTLA‐4 promoter methylation was shown to be a viable biomarker for the efficacy of immune checkpoint blockade specifically in melanoma and subsequently in renal cell carcinoma (ccRCC).^12^ By using mRNA to analyze the methylation gene, PD‐L1 expression, and CD8^+^ T cell infiltration of patients, it was shown that hypomethylation of the CTLA‐4 promoter showed to be a strong biomarker for poor prognosis in patients with ccRCC at initial diagnosis. However, the hypomethylation of CTLA‐4 promoter biomarker was found to have favorable outcomes in renal cell carcinoma patients treated with immune checkpoint blockade. When evaluating a combinatorial approach of anti‐CTLA‐4 and anti‐PD‐1, patients treated nivolumab‐plus‐ipilimumab group had a survival rate of 52%, while a 44% for nivolumab only and 26% for ipilimumab only group in a 5‐year period.^13^ These therapeutics do present with side effects associated with increased immune activation. With anti‐PD‐1 treatments, patients experienced thyroid dysfunction, type 1 diabetes mellitus, and pneumonitis; while colitis, diarrhea, hypophysitis, and adrenal insufficiency are more associated after anti‐CTLA‐4 treatment.^14^

In addition to T cells, natural killer cells (NK) participate in surveillance of the body for “altered self” cells. If their inhibitory receptors fail to recognize a cell as “self,” NK activation will commence. Upon activation, NK cells release cytotoxic granules containing perforin and granzymes to lyse the cells. This is done without any priming or prior activation. NK cells are also a large producer of cytokines‐interferon gamma (IFNγ) and TNFα, which enhance the immune response of dendritic cells and macrophages. However, tumor microenvironments have the capability of releasing ligands that activate NK inhibition by binding to the NKG2A receptor.^15^ Studies have shown that melanoma patients have exhibited reduced cytotoxicity in NK cells. However, when memory‐like differentiation has occurred in NK cells from both healthy donors and melanoma patients, there was an increased production of IFNγ in both populations.^16^ Results like these have opened the door for using NK cells for immunotherapy.

#### 1.1.4 Engineered cell therapeutics

To target tumors using NK cells as a therapeutic, NK cells are generated from peripheral blood as well as pluripotent stem cells. They are then expanded and activated with the use of IL‐2, IL‐15, and other cytokines to increase their cytotoxic capabilities and then administered into patients. Implementation of autologous NK cells in clinical studies has not been very successful in tumor reduction.^17^, ^18^ Due to some clinical limitations of NK cell‐only therapy, engineered human NK cells derived from pluripotent stem cells have also been explored. An in vivo mice study investigated the modification of pluripotent stem cells derived from NK cells that had a high affinity to the non‐cleavable version of CD16 receptor. This experiment showed by treating lymphoma with the combination of the modified NK and anti‐CD20 mAb resulted in the reduction of solid tumors that were previously resistant to NK treatment.^19^

**FIGURE 2:**
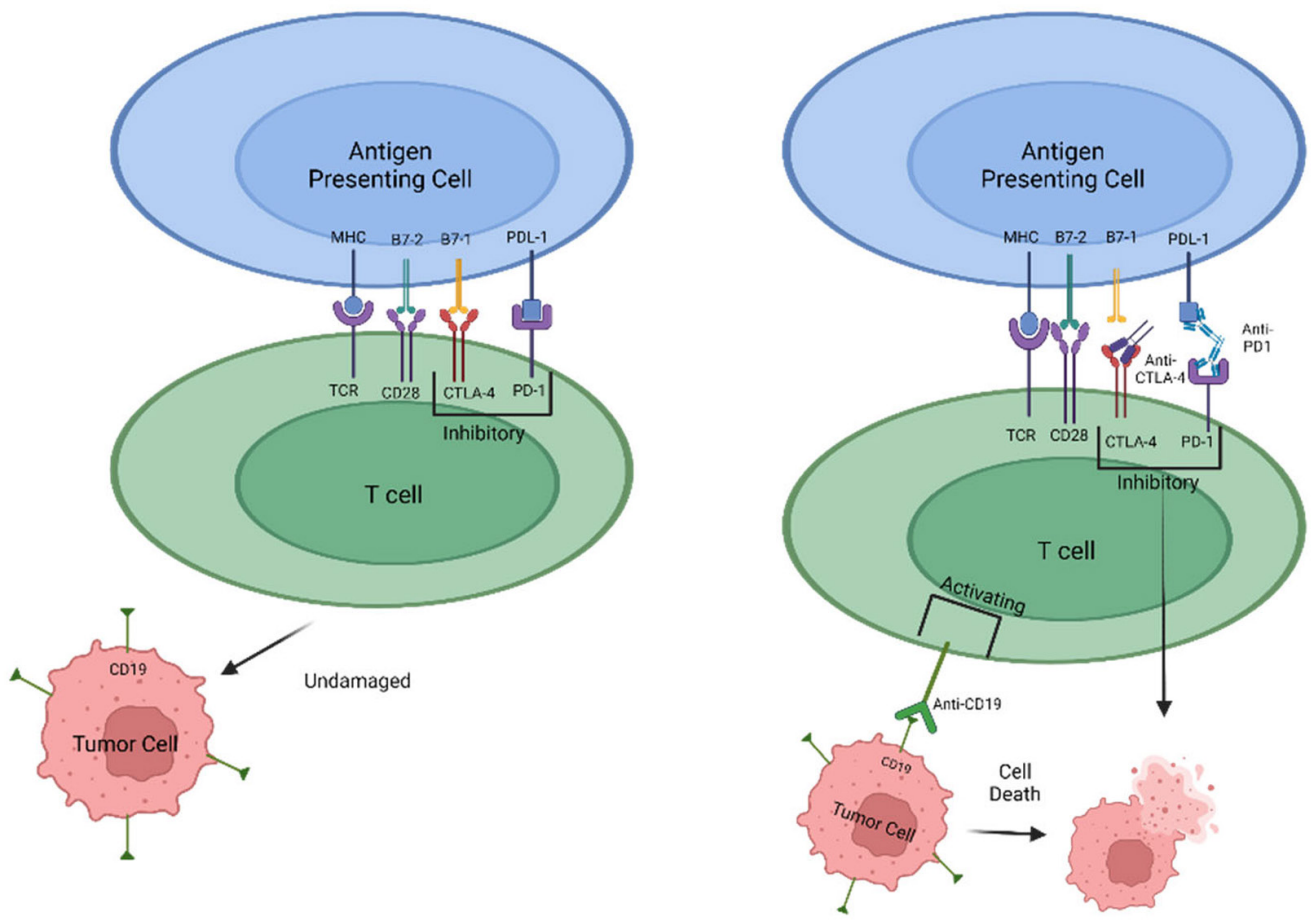
Mechanisms of inflammatory response through genetically modified T‐cells. Recognizing stress‐induced ligands on cancerous cells allowing NK and T cell activation to cause cell death of cancer cells via CAR‐T therapy. Inhibitory binding mechanisms via anti‐PD‐1 and anti‐CTLA‐4 can also cause apoptosis of cancerous cells. Modified from “Cancer Mechanisms,” by BioRender.com (2023). Retrieved from BioRender.com.

In addition to NK cell therapy, cytotoxic T lymphocytes (CD8s) have been targeted for their ability to specifically detect cells with “altered self.” As a result, chimeric antigen receptor (CAR)‐engineered T‐cell (CAR‐T) is another avenue of cancer immunotherapy being investigated.^20^ T‐cell receptors are genetically modified to recognize certain antigens on cancer cells. They have been shown to be effective in dealing with hematological malignancies but there are current issues with dealing with solid tumors due to the possibility of cytokine release syndrome, poor homing, and trafficking into solid tumor sites, and the immunosuppressive qualities of the tumor microenvironment. The cells recognize stress‐induced ligands on tumors to allow the activation of T cells and NK cells. This led to the FDA approval of tisagenlecleucel (Kymriah) in 2017, a CAR‐T therapy based on the modification of CD19 for patients under 25 years old with relapsed B‐cell non‐Hodgkin lymphoma (Figure 2).^21^

#### 1.1.5 Nanoparticle‐based immune activation in cancer therapeutics

While the use of antitumor vaccines has transformed cancer treatments, some have been limited in their ability to produce a strong immune response. In addition to targeting cells themselves as therapeutics, researchers have investigated synthetic methods for immune stimulation through engineered carriers to allow for more fine‐tuned drug delivery both in space and time. This has led to the development of nanoparticle (NP)‐based vaccines (nano vaccines). They work by activating immune cells through receptors that have been shown to play a role in anti‐tumor immunity. The size, material formation, and surface charge are important to the pharmacokinetics and ability of these nanoparticles to activate the immune system. In vitro, a non‐toxic concentration of 10 nm gold nanoparticles compared to 50 mm significantly impaired the activation of human dendritic cells (DC) by lipopolysaccharide‐induced maturation by impairing the upregulation of CD83.^22^ Researchers using poly (lactic‐co‐glycolic acid) (PLGA) showed a greater activation of DC with nanoparticles of 300 nm than 17, 7, and 1 μm. When evaluating the size dependence of PLGA nanoparticles engulfed by macrophages it showed small particles such as 389 nm were phagocytosed producing an inflammatory response while those 6.5 μm remained on the surface of the macrophages at the time of analysis.^23^, ^24^, ^25^ In addition to size, the chirality of nanoparticles is an important aspect of its interaction with the immune system. Chiral nanoparticles, specifically l‐type as opposed to d‐type, were analyzed and found to be capable of initiating the innate and acquired immune response in mice. In murine models, an upregulation of CD8+ T cell infiltration and NK cell activation were detected in nanoparticle treatment versus control models.^26^

Nanoparticle vaccines have the capability to induce an immune reaction within the body. One of the methods is through the stimulator of interferon genes (STING) pathway, which detects DNA in the cytoplasm through downstream signaling activated type I interferon beta (IFN‐β). It has been shown that STING activation can induce pro‐apoptotic molecules such as BAX, Puma, and Noxa in T cells.^27^, ^28^ In previous studies, it has been shown that mice deficient in the STING pathway experienced rapid tumor growth and had impaired cytotoxic T cells.^29^, ^30^ Immune activation via nanoparticles carrying STING agonists has shown promise in pre‐clinical studies. Lipid nanoparticles with heterocyclic lipids that stimulate STING signaling and deliver tumor antigens simultaneously have been proposed as a potential cancer vaccine platform.^31^ Pulsatile‐release particles loaded with STING agonists have been employed in intertumoral injections for sustained inflammatory signaling inducing immune responses and tumor regression in mice.^32^ In other cases, nanoparticles have been developed to rupture when encountering the acidic environment of the lysosome resulting in activation through MHC‐I antigen processing.^33^ In an in vivo study using *α*‐Al_2_O_3_ nanoparticles, cross‐presentation of antigens was improved thereby improving the efficacy of tumor cell‐derived autophagosomes soluble ovalbumin. It was shown that cross‐presentation was increased through the engagement of the autophagy pathway and ultimately led to the rejection of tumor rejection for 40 days within the mice species.^34^

Nanoparticle immunotherapies can be modified to change the kinetics of drug delivery through changes in surface coatings and nano/microparticle structure. It has long been appreciated that addition of polyethylene glycol (PEG) polymer chains on the outside of particles extends their circulation half‐life through modifying the adsorbed protein corona.^35^ Furthermore, as referred to previously, through generating particles that have a burst release as opposed to a slow‐release profile, scientists have been able to create single‐injection vaccines for delivery of STING agonists.^32^ Biodistribution of nanoparticles and their pharmacokinetics have been evaluated extensively with different material types and surface coatings.^36^

Through understanding the specific mechanisms of immune activation, and how those are manipulated in tumors to evade immune detection, researchers have been able to engineer therapeutics to help treat cancer both through small molecule drugs, protein drugs, and cellular therapeutics. Each of these therapies has different considerations in terms of cost, delivery, and side‐effects that will be improved with further work on both basic immunology and engineering new therapies for these targets.

### 1.2 Regenerative medicine

While cancer immunotherapy seeks to activate immune cells to destroy altered‐self (malignant cells), immunotherapies for wound healing must also stimulate immune reaction to damaged self while promoting repair and regeneration as opposed to malignant cell killing. Acute and chronic wounds affect millions of individuals worldwide annually and in the United States alone on average can cost a patient around $15 000 leading to clinical and economic burdens.^37^, ^38^ In comparison to funding allocations, resources, and advertisements for cancer research, there is not as substantial support for wound healing^39^; thus, making it even more imperative to bring attention to the deficit in therapeutics for wound healing outcomes to advance clinical outcomes as the molecular mechanisms of wound healing are closely associated to the dysregulated generation of cancer tissue.^4^

#### 1.2.1 Wound healing therapeutics

As multiple factors can inhibit or deter wound healing outcomes, some wounds can heal without intervention, while others cannot. In cases where an acute wound takes longer than 4–6 weeks to close, it persists into a chronic wound, further impeding the quality of life for these individuals. It is imperative that wounds heal completely within a reasonable time frame to increase the quality of life for those who suffer from an infection and may experience multiple cases of chronic wounds in their lifetime, such as diabetic, obese, or elderly patients. Almost 5% of the United States population is affected by chronic wounds, but the percentage is much higher within at risk groups.^40^, ^41^, ^42^ Additionally, chronic wounds require a lot of maintenance surrounding their wound dressings, and the potential risk of infection increases the longer the wound is open and exposed to the outside environment, in addition to a higher stress response in patients. As stress is linked to a cortisol hormone increase in the bloodstream, this can further deter wound healing through immunosuppression, as well as potential mobility loss depending on the wound site location, disturbed sleep patterns, and in some cases development of depression.^43^ As such, decreasing the healing time and improving the quality of resulting tissue is the target of clinical practice. One important approach in the clinic is maintaining the moisture barrier as to not excel bacterial growth with excess moisture but to maintain enough moisture to promote healing and prevent further injury and edema.^44^ As the immune system is the driving force in the wound‐healing process, basic immunological perspectives are widely considered in wound‐healing approaches.^45^ Often, there are a number of different pharmaceutical interventions that occur during the course of wound healing, including antibiotics, steroids, and biologics to promote closure and recovery. Delivery of these therapeutics to the wound space and its desired kinetics depend on the tissue that is injured, but often these cases center around skin wounds.

Overall, there are four general stages of wound healing.^46^ The first is the hemostatic and clotting phase where red blood cells, platelets, and a provisional matrix generate a clot to prevent excessive bleeding which in turn signals to begin the immune response. The second stage is the inflammatory stage which on average lasts around 4 days and is the acute wound phase as this recruit immune cells to fight off the infection, clear out debris, and provide important cues for subsequent tissue development. The inflammatory stage overlaps partially with the proliferative phase as this is when fibroblasts proliferate, begin to deposit collagen, and repair the damaged tissue. The remodeling stage differs between chronic and acute wounds as in severe cases this stage can last months leading to either lack of closure or hypertrophic scar tissue development if the wound does not heal properly. Some therapeutic approaches consider inhibiting inflammation specifically during the remodeling phase via immunosuppressive drugs^47^; however, by delaying inflammation during the first phase of wound healing, this could lead to excessive bacteria growth, infection, or even sepsis in some cases as immune cell recruitment during early phases of wound healing are of the essence to benefit patient outcomes.^48^ As much as the inflammatory stage is helpful, it is essential that this is not prolonged as to not contribute to a systemic immune response, which is why the maintenance and regulation of the inflammatory phase to the proliferative phase is imperative.^49^ As the first cell recruitment upon wound injury is neutrophils, research led to the investigation of neutrophils on further downstream immune cell recruitment and interestingly found that the absence of neutrophils did not alter macrophage phenotype. These findings led to investigations on macrophage modulation and how their expression can trigger an immune response and alter wound healing outcomes, especially during a prolonged inflammatory phase such as chronic wound cases.^50^

Diabetic patients who suffer from systemic pro‐inflammatory issues and chronic inflammatory cases such as diabetic foot ulcers (DFU) can end up with amputations if not caught and treated early on. For these patients, DFU and chronic wounds were shown to have a delayed healing outcome when *FOXM1* was inhibited, further decreasing the recruitment of immune cells and can in turn change an acute wound to a chronic wound state in these patients as *FOXM1* and associated proteins were decreased in DFU patients.^51^ As these patients have a prolonged perpetuated inflammatory state due to the non‐enzymatic glycation of lipids and proteins there is continual immune cell recruitment leading to downstream effects of reactive oxygen species (ROS), and growth factors such as TCF‐β, and inflammatory cytokines deterring the proliferative phase from taking place. Also the hyperglycemic environment inhibits the M1‐to‐M2 transition of macrophage which delays and prolongs the inflammatory stage as such investigations center on immunomodulation early in the wound healing phase.^52^ This has led to clinical studies collecting blood from patients with and without DFUs to study TCR‐αβ+ T‐cell subpopulations. This study investigated differences in TCR‐αβ+ T cell populations from diabetic patients who had acute and chronic DFUs to determine if T‐cell subpopulation concentrations differed in non‐DFU cases and found an overall lower diversity of CD4+ T cell populations in diabetic versus non‐diabetic patients.^53^ Identifying these differences between normal and diabetic wound healing can be leveraged in immunomodulatory therapeutic design and further help wound healing outcomes. These findings led to research conducting phase one clinical trials in type‐1 diabetic patients utilizing polyclonal T_reg_ infusions to modulate the immune system and effectively expressed CD45RA T_regs_. This study found that T_reg_ function improved overall, with potential benefits in their disease course (protecting pancreatic islets) further serving as a potential therapeutic target in diabetic patients.^54^

Centering immunomodulation with therapeutic design can also be incorporated with wound dressings such as hydrogels and incorporation of immune cells or growth factors to modulate the immune microenvironment. Stem cell therapies have also been investigated for their proliferative and pluripotency potential and have been found to be more beneficial in comparison to skin grafts. Stem cells can also benefit diabetic patients or those who suffer from autoimmunity issues in general as they can be differentiated into the needed cell type and can be leveraged in the regenerative medicine field. Experiments showing the benefits of stem cells specifically in immune‐compromised patients, have led to therapeutic inquiry utilizing stem cell subtypes.^55^ A common stem cell such as mesenchymal stem cells, function as immunomodulators via communication networks between varying immune cells,^56^ this knowledge led to studies using adipose stem cells in murine models to test and increase vascularization and further induce collagen production in burn wounds. Another study utilized amniotic stem cells to promote regeneration of epithelial cells, further contributing to beneficial therapeutic designs for diabetic patients and wound healing.^57^, ^58^

**FIGURE 3:**
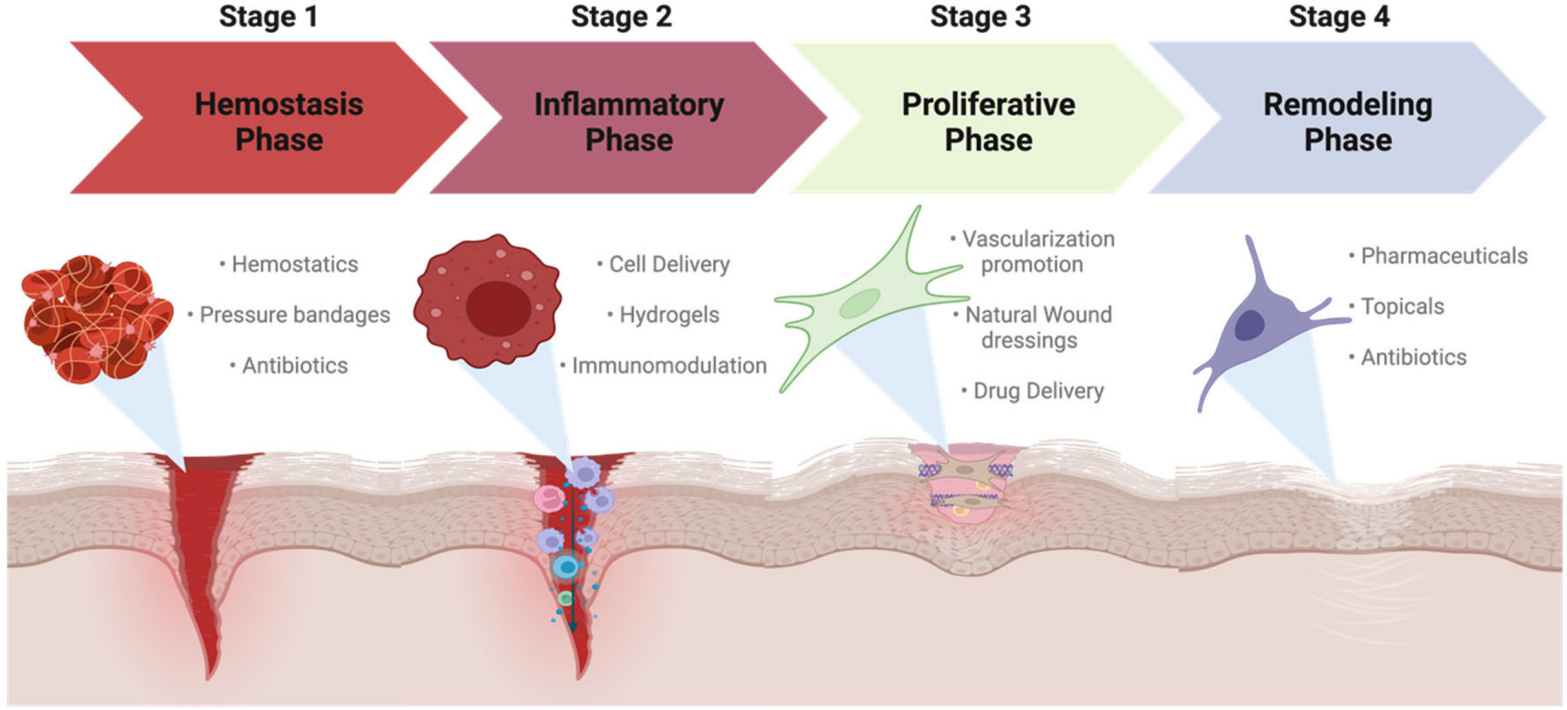
Wound healing phases and therapeutic treatment options throughout the following healing stages. Targeted treatments during the first inflammatory phase of wound healing consist of using differing biomaterials and hydrogels to cover the wound and novel T_reg_ Infusions to alter immune cell population. During the proliferative phase, drug delivery systems and nanoparticles are used to deliver growth factors and stem cells to influence proper wound healing. The final remodeling stage is typically treated with pharmaceuticals and antibiotics if needed, but novel wound dressings are used to ensure the wound healing process completes successfully and the wound does not re‐open. Modified from “Wound Healing,” by BioRender.com (2023). Retrieved from BioRender.com.

As biocompatibility of wound care devices is another concern for patients suffering from chronic wounds, wound dressings that do not illicit strong inflammatory reactions are strongly needed in the clinic, especially since they provide a protective barrier from the outside environment and the wound to prevent further inflammation and infection. Approaches currently being investigated have grown far beyond the original cotton gauze dressing and consider many factors such as optimal coverage for wounds, maintenance of the moisture barrier, and even biomaterial interactions and cellular signals such as growth factors and biologics to promote wound healing in the most optimal time possible. Pro‐regenerative biomaterials such as extracellular matrix (ECM) have been found to reflect an alternatively activated (M2) macrophage phenotype leading to beneficial healing outcomes.^59^, ^60^, ^61^, ^62^ With this immunomodulating knowledge, research has been done to incorporate these factors into wound healing treatment. These approaches ensure that the pharmacological, pharmacokinetic, and mechanical requirements of wound healing are met to provide a novel approach to promote the healing process and increase the quality of life for those who suffer from chronic wounds (Figure 3).^63^

Not only do we need to focus on the healing aspect of wounds, but also to avoid adverse reactions to treatment so the affected area is functional, depending on wound size, area, and location. This is where engineers have utilized biomaterials to advance wound healing outcomes and target the immune response to promote regeneration of the injured tissue leading to a chronic wound. In cases of biomaterial treatment, biomaterials can be broadly classified as either natural or synthetic based materials. Natural biomaterials tend to have less of a standard, immunogenic, foreign body response (FBR) in vivo than synthetic materials.^64^, ^65^ As such, they have been applied for patients suffering from chronic wounds as they are more at risk of a fibrotic reaction leading to scar tissue development than functional, healthy tissue after a wound closure.

From an immunological perspective, as certain growth factors, cytokines, and cells play an integral part in the wound healing process, investigations surrounding the incorporation of these factors with biomaterials to assist not only the wound healing process but tissue regeneration as well. Within the phases of wound healing, various growth factors and cytokines can help promote the progression of the wound to the healing phase.^66^, ^67^, ^68^ In cases of chronic wounds that are delayed in the inflammatory phase, the absence of cells that produce and secrete the required growth factors and cytokines or the degradation of those that are present can determine how long the wound takes to reach the healing phase.^69^ In order to control these different factors, engineers have employed a variety of different materials to control drug delivery and biodistribution,^1^ focused both on previously defined targets such as antibiotic delivery and new targets that have been discovered from the evaluation of immunologic role in different tissue regeneration pathways.^70^

Utilizing hydrogels and microspheres to promote wound healing outcomes has become a highly investigated strategy. Hydrogels act as a multifactorial therapeutic as they can be used for wound dressings and bioactive drug delivery by promoting a hydrated environment and forming a barrier from pathogens while delivering necessary pharmaceuticals.^71^ Hydrogels can be loaded with growth factors and antibiotics in cases of persistent infections.^72^, ^73^ Researchers have shown the concurrent use of biomaterials, antibiotics, and growth factors to help promote wound healing outcomes. A study led by Huang, et al. combined two common natural biomaterials to make a hydrogel backbone with chitosan and hyaluronic acid to deliver a popular clinical antibiotic‐vancomycin.^74^ This is advantageous as the hydrogel formed in situ, potentially serving as a therapeutic option for wound closure simultaneously promoting an antibiotic microenvironment directly at the injury site as well as hydrogels crosslinked with gentamicin further promoting prolonged pH‐reactive antibiotic diffusion to the wound site.^75^ As these studies were in murine models, further clinical studies would need to be conducted for translational purposes. By encapsulating antibiotics and growth factors such as vascular endothelial growth factor (VEGF)—a signaling molecule to help rebuild vascular tissue in PLGA microspheres can provide optimal therapeutic options for wound healing and regeneration of lost or damaged tissue. Beyond microspheres, VEGF mRNA has also been encapsulated in an ionizable lipid‐mediated nanoparticle via microfluidics to express this protein and benefit wound healing in diabetic patients, although this has only been evaluated in murine models thus far.^76^ Incorporating immune cells, growth factors, and pro‐regenerative biomaterials is crucial for beneficial wound healing outcomes, especially in diabetic patients. In a typical wound healing process immune cells interplay with metabolic processes and when these processes are impacted in diabetic patients, wound healing can be deterred causing more issues down the line for patient recovery. Overall, as we have seen innate immune cell processes being widely impacted when triggered in a diabetic wound model, immune cells interact with damage‐associated molecular patterns and if the inflammatory phase is activated for too long these become chronic cases further leading to the target of immunomodulating materials for treatment.^77^

With translational pursuits in mind, diabetic wound repair is a common wound that is investigated and can benefit highly from multifunctional natural‐based hydrogels. To further promote a beneficial wound healing outcome, natural biomaterial‐based hydrogels can even be loaded with plant‐based compounds such as curcumin to promote angiogenesis further without using a growth factor, potentially serving greater accessibility in clinical settings.^78^, ^79^ In addition to tissue‐derived products such as collagen‐based biomaterials, researchers have also investigated plant‐based products. Recently, a novel bioengineered FDA‐approved wound product made of hydroxyapatite, collagen, and Manuka honey, has helped diabetic wound healing outcomes and promoting an anti‐bacterial environment.^80^, ^81^ Current applications being investigated surrounding wound repair include biocompatible patches and hydrogels as therapeutic targets. Beyond skin wound repair, patches can be manufactured to help repair the regeneration of lung tissue in cases of puncture wounds and serve as another viable option for potential therapeutic treatment in regenerative medicine.^82^

#### 1.2.2 Medical devices coatings and implantation

Newly engineered therapeutics being developed for re‐establishment of tissue function range beyond wound healing. Research currently being investigated surrounding medical devices, coatings, and implantations is centered around limiting the body's adverse reactions to these treatments which is a similar approach as discussed with wound healing outcomes. By directly administrating targeted therapeutics to the source (the implanted device), further acceptance of the implanted material or device can benefit patients in multiple clinical outcomes. Medical devices can be coated with pharmaceuticals to limit the body's adverse reaction to a foreign body material and to increase biocompatibility of the device.^83^ As biomaterials and medical devices are broadly used in prostheses and implants, the introduction of an implant into a soft or hard tissue induces a temporary inflammatory reaction followed by tissue repair and scarring around the implant. This investigation of material implantation takes a different immunological approach for integration compared to wound healing targeted treatments. Research conducted on the prolonged inflammatory response after implantation focuses on improving tissue integration and subsequent long‐term maintenance through modification of the device or implant surface via mechanical, chemical, or biological functionalization. Modifying these properties is a critical target of interest as implantation of devices in vivo elicits a FBR to the implant.^84^, ^85^

The main issue surrounding medical devices, coating, and biomaterial implants, is the immune‐mediated reaction known as the FBR. Upon implantation, the adjacent tissue becomes injured, causing a strong innate inflammatory response as the body does not recognize the device or implant as self, further leading to downstream events as well as adaptive immune cell recognition.^86^ If the body is unable to degrade the material through oxide radicals and enzymatic secretions, there is an accumulation of collagen deposition at the material surface forming a fibrotic encapsulation surrounding the medical device or implant. This strong inflammatory reaction can lead to a reduced function of the device or failure or material rejection. Reducing or limiting the FBR has been the focus of designing next‐generation medical devices and implants to decrease adverse reactions patients experience and incorporate more biocompatible materials to elicit a tolerogenic and pro‐regenerative macrophage phenotype in comparison to a pro‐inflammatory phenotype.^87^ Different material structures, shapes, and coating materials such as zwitterionic hydrogels composed of poly (carboxybetaine methacrylate) with a carboxybetaine monomer (CBMA) and a carboxybetaine cross‐linker (CBMAX) have been investigated to reduce adverse reactions for the patient by resisting non‐specific protein absorption as well as larger sized hydrogels of differing materials were able to avoid the FBR, but the problem remains a clinical concern.^88^, ^89^

Researchers have utilized several approaches to coat medical devices thoroughly to suppress fibrotic encapsulation, and in turn, reduce the FBR that is elicited upon material and device implantation. The body typically has a stronger reaction to implants or medical devices made of synthetic materials than natural materials, though there have been advances in modifying synthetics. Synthetic material types include materials such as metals, inorganics, and natural or synthetic polymers and are widely used due to their strength and long‐term durability. As the perfect material does not exist, the goal is to achieve a durable, more biocompatible material for overall incorporation into the body. Although more biocompatible materials have been incorporated such as extracellular matrix, polyethylene glycol, poly (2‐hydroxylethyl methacrylate), polyethylene, and crosslinked collagen, the FBR continues to be a concern.^90^, ^91^ Some studies have used coating materials such as biodegradable polymer poly lactic‐co‐glycolic acid and polydioxanone to try and incorporate anti‐fibrotic and anti‐inflammatory pharmaceuticals to reduce the FBR.^92^ Work done by Pakshir et al. coated devices with corticosteroids to deter device encapsulation and promote device integration for long‐term use in vivo.^93^ They also coated these materials on silicone discs as it is a common material implant used in clinical settings that typically elicits fibrotic buildup and collagen deposition at the material surface. Their first material coating was dexamethasone (Dex) combined with triethylene glycol (Teg) and Dex combined with 1,6‐hexanediol (Hex). By comparing different molecular weights of these biomolecules, their goal was to have different release rates of Dex to dissolve this coating from the implant surface at the slowest release rate possible to maintain device compatibility long‐term. This study found in vitro drug release of Dex‐Teg‐Dex was released in a shorter duration than Dex‐Hex‐Dex, which can potentially be a beneficial therapeutic for long‐term maintenance of medical devices. After 7 days, the slow‐release profile of both combinations effectively reduced the number of CD68‐ and MHCII‐positive macrophages at the tissue‐implant interface further promoting a pro‐regenerative macrophage polarization phenotype. This work provides a beneficial therapeutic option for medical device protection from an acute inflammatory response and incorporates the use of natural biomolecules that are biocompatible over inflammatory‐prone polymeric drug delivery systems, which also incur problems surrounding drug loading capacities, further limiting drug exposure to the surrounding tissue of the implant.

In addition to dexamethasone coatings, other immunopharmacologic agents such as FK506 (tacrolimus, a T cell inhibitor) and rapamycin (an mTOR inhibitor) have been used to specifically dampen immune responses to medical device implants.^94^, ^95^ While promising in comparison to untreated materials, any systemic applications of FK506 and rapamycin could lead to immunosuppression in the patient that would lead to the potential for opportunistic infections. At the implant site, the immune system is needed to assist in defense against bacterial infections. Once a material is infected, it often has to be excised, then an extended period of recovery of the implant site before trying to re‐implant due to damage to local tissue. As such, broad immunosuppressive agents are less desirable than some specific and local therapeutics.

To avoid systemic immunosuppressive effects, in a top‐down engineering approach, researchers have also screened a library of different biomaterial surface modifications that involve covalently linked functional groups to modify the interaction of the body with an implanted material. One recent example includes modification of alginates with triazole groups that inhibit fibrosis of alginate in the peritoneal cavity in both mice and non‐human primates.^96^ This was also verified in the subcutaneous space of mice and was correlated with alterations in immune cell function. Other surface modifications have been used on polymeric devices, such as those made of polydimethylsiloxane (PDMS), that prevent fibrotic encapsulation and suffocation of transplanted pancreatic islets.^97^ While promising therapeutics, their mechanism of action is still unknown, which decreases the ability to optimize these materials and screen candidate alternatives based on the immune function that is being targeted by the drug.

Other approaches in modulating the immune system consist of using corticosteroids, gene delivery via lentivirus, or scaffold integration of cytokines to alter macrophage polarization to promote a regenerative healing and implant integration phenotype.^98^, ^99^ Even specific cellular engineering has been done on DC modulation to further modulate the immune system via polydopamine.^100^ Overall immunomodulation for device and implant acceptance have advanced in the field and with the use of pharmaceuticals and biological markers can aid engineering targets for further therapeutic designs for host integration and regeneration.

## 2 DISCUSSION

Investigating the immune response to cancer treatment, wound coating materials, and implants, can further serve as a potential tool to target more biocompatible therapeutic options. Using a bottom‐up approach allows investigators to understand the immune microenvironment and fine‐tune treatment options based off cellular signaling.

In specific cases of oncolytic viruses, immunologists can look through interactions with TLRs to assess molecular patterns of signaling cascades and inflammatory molecules that are triggered by the virus. By understanding the basic biological cues occurring from the oncolytic virus response, T‐VEC was developed to be used in melanoma treatment, further broadening the possibilities of treating illness and diseases through this approach and shedding light on immunotherapy. Further focusing on specific immune cells, such as T cells and NK cells have been investigated highly in the immunotherapy field as they can activate cytokines and other inflammatory markers to help the body fight off tumors, as tumor microenvironments evade immune recognition by the release of ligands that inhibit T and NK cells. Murine studies have utilized NK cells derived from human pluripotent stem cells along with anti‐CD20 mAb to treat lymphoma. In addition to checkpoint blockade immunotherapy that removes these brakes that tumors put on immune cells, researchers have also developed chimeric antigen receptor (CAR)‐modified T‐cell (CAR‐T) therapy to recognize certain tumor antigens and specifically target cancer cells. As multiple FDA approved CAR‐T (ex. Anti‐CD19 CAR‐T, for B cell leukemias and lymphomas) are now used in clinical settings, targeted immunotherapy outcomes are more prevalent than ever.

Wound healing and regenerative medicine therapeutics in comparison to cancer immunotherapy investigations are less investigated due to regenerative medicine being a newer field of study and newly appreciated as having a strong immune component. However, therapeutics for chronic wounds are on the rise. With the improvement of wound coverings over time, more therapeutic options are presenting with the use of biomaterials, microspheres, hydrogels, and even the addition of natural components to further help wound healing outcomes. With wound healing, treatment is largely targeted during the inflammatory phase as this is when cytokines and growth factors are released at the site of injury. By utilizing biocompatible natural biomaterials, growth factors, and other molecules, wound healing outcomes in chronic wounds have had an improved healing rate which further provides a better quality of life for patients who experience these chronic cases often.

In terms of biocompatibility, medical devices and implants can struggle to find acceptance from the body and in turn are rejected by the host, often causing damage to the surrounding tissue. To reduce the FBR response caused by this implanted device/material, surface coatings have been utilized to effectively incorporate the foreign device into the host for long‐term biocompatibility and maintenance. By incorporating immunomodulatory pharmaceuticals, targeted cell therapies, and incorporation of biologics to alter macrophage polarization and cytokines for example into the implant as well as treatment with antibiotics, steroids, gene delivery/inhibition, and cellular engineering has shown success in mitigating this response.

By understanding basic biological processes in response to cancer, implants, or damaged tissue from wounds we can further target therapeutic designs to achieve a higher patient acceptance and recovery rate. From an immunological perspective, there is no clear‐cut way to target differing illnesses, diseases, or reactions, but for example, by understanding one regulatory mechanism such as wound healing and regenerative patterns associated with this healing process, this knowledge can be incorporated to target cancer treatment as it is a misguided regulatory mechanistic process. By investigating these clinical issues with a bottom‐up approach to target therapeutics, we can understand molecular mechanisms and cell signaling patterns to design therapeutics based off the mechanisms and incorporation of pharmacologics to build on sustainability long‐term and incorporate overall patient satisfaction.

Recently, there have been advances in our understanding of diseases once thought to be unrelated to immunology but possess a strong immune element. Several neurologic diseases including multiple sclerosis have been correlated with prior viral infections. This opens a door for potential vaccination to prevent downline pathology, just like vaccination against HPV (found to be a causative agent of cervical and head and neck cancers) was associated with a decrease in cancer incidence. Extreme examples of a lack of knowledge about a system leading to improper therapeutics can be seen as far back as using leaches to draw out “toxic blood” but even in present day where women go undiagnosed for years before finding they have severe endometriosis. As we learn more about our immune system and how it is regulated, we can then target those pathways for therapeutics.

## 3 NOMENCLATURE OF TARGETS AND LIGANDS

Key protein targets and ligands in this article are hyperlinked to corresponding entries in http://www.guidetopharmacology.org, the common portal for data from the IUPHAR/BPS Guide to PHARMACOLOGY (Harding et al., 2018),^101^ and are permanently archived in the Concise Guide to PHARMACOLOGY 2019/20 (Alexander et al., 2019).

## AUTHOR CONTRIBUTIONS

S.D. and D.F. generated the figures. S.D., D.F., M.F., and K.S. performed literature reviews and wrote the manuscript. K.S. edited and revised the manuscript.

## Conflict of Interest STATEMENT

The authors declare no conflict of interest.

## ETHICS STATEMENT

No experimentation was performed to generate this review and all information displayed are from publicly available journal articles retrieved through applicable subscriptions via the NIH Library.

## Data Availability

No original data were generated in this review article. All data cited are from published works.
